# Efficient perovskite solar cells based on low-temperature solution-processed (CH_3_NH_3_)PbI_3_ perovskite/CuInS_2_ planar heterojunctions

**DOI:** 10.1186/1556-276X-9-457

**Published:** 2014-09-02

**Authors:** Chong Chen, Chunxi Li, Fumin Li, Fan Wu, Furui Tan, Yong Zhai, Weifeng Zhang

**Affiliations:** 1 School of Physics and Electronics, Henan University, Kaifeng 475004, People’s Republic of China; 2 People’s Republic of China and Henan Key Laboratory of Photovoltaic Materials, Henan University, Kaifeng 475004, People’s Republic of China; 3 Institute of Modern Physics and School of Science, Huzhou Normal University, Huzhou 313000, People’s Republic of China

**Keywords:** Solution-processed, Solar cells, (CH_3_NH_3_)PbI_3_, Perovskite, CuInS_2_

## Abstract

In this work, the solution-processed CH_3_NH_3_PbI_3_ perovskite/copper indium disulfide (CuInS_2_) planar heterojunction solar cells with Al_2_O_3_ as a scaffold were fabricated at a temperature as low as 250°C for the first time, in which the indium tin oxide (ITO)-coated glass instead of the fluorine-doped tin oxide (FTO)-coated glass was used as the light-incidence electrode and the solution-processed CuInS_2_ layer was prepared to replace the commonly used TiO_2_ layer in previously reported perovskite-based solar cells. The influence of the thickness of the as-prepared CuInS_2_ film on the performance of the ITO/CuInS_2_(*n*)/Al_2_O_3_/(CH_3_NH_3_)PbI_3_/Ag cells was investigated. The ITO/CuInS_2_(2)/Al_2_O_3_/(CH_3_NH_3_)PbI_3_/Ag cell showed the best performance and achieved power conversion efficiency up to 5.30%.

## Background

Thin-film solar cells have attracted considerable attention because of simplified and low-cost fabrication procedures compared to conventional silicon-based solar cells. The thin-film solar cells based on inorganic photovoltaic materials processed with expensive vacuum-based techniques and/or high-temperature sintering exhibit high efficiency [1-3]. However, the use of these thin-film solar cells is still limited because the manufacturing costs are still relatively high. To lower the cost of device fabrication, the low-temperature solution-based techniques such as spin coating and chemical bath deposition are needed to prepare inorganic photovoltaic materials. The thin-film solar cells based on the solution-processed inorganic nanocrystals such as PbS [4,5], CdTe [6,7], CdSe [8], copper indium disulfide (CuInS_2_) [9,10], and Cu_2_ZnSnS_4_[11] have been demonstrated, but their maximum solar power conversion efficiency is still low. The main reason for the low efficiency is that the low-temperature solution-processed inorganic nanocrystals are typically amorphous or poorly crystalline, leading to poor charge carrier transport because of short carrier diffusion lengths (typically about 10 nm). Therefore, the new solution-based techniques to improve the crystalline structures of inorganic nanocrystals are needed. For example, to enhance the carrier transport in CuInS_2_, a method of using a molecular-based precursor solution has been presented [10] to synthesize CuInS_2_ nanocrystals with a polycrystalline structure at relatively lower temperatures (<250°C) for the solution-processed inorganic solar cells. Besides, the inorganic materials which can be processed with solution-based techniques and generated charge carriers with long diffusion lengths in the bulk are sought.

The recently reported semiconducting perovskite materials such as (CH_3_NH_3_)PbX_3_ (X = Cl, Br, I) could fulfil these requirements. These perovskites have high charge carrier mobilities and long charge carrier lifetime, which means that the light-generated charges have long carrier transport lengths [12]. It has been reported that the effective diffusion lengths are about 100 nm for both electrons and holes [13,14]. In addition, these perovskites with a direct bandgap have a broad range of light absorption and high extinction coefficient [15,16]. Due to their super electrical properties and super light-harvesting characteristics, the perovskites have been used in a variety of nanostructured solar cells and have achieved high-power conversion efficiencies (>9%) [16-20]. In solid-state sensitized solar cells, the CH_3_NH_3_PbI_3_ used as the sensitizer has led to a high-power conversion efficiency of 15% [17]. The other perovskite-based nanostructured solar cells that commonly incorporated the perovskite as the absorbing layer between an n-type electron-transporting layer such as TiO_2_ and a p-type hole-transporting layer such as 2,2′,7,7′-tetrakis(N, V-di-p-methoxyphenylamino)-9,9′- spirobifluorene (Spiro-OMeTAD) have also demonstrated high efficiencies [15,18,21]. Moreover, the research results reported by Lee et al.[16], Etgar et al.[22], and Ball et al.[23] showed that the perovskites have good charge (electron or hole)-transport properties, resulting in high efficiencies of the solar cells. Nevertheless, in these perovskite-based nanostructured solar cells, the transparent TiO_2_ compact layer between the conducting substrate and perovskite materials or the scaffold (Al_2_O_3_) generally requires high-temperature sintering at about 500°C [15,17,18,23,24], which limits substrate choice and is incompatible with the low-cost solar technology. Therefore, the low-temperature solution-processed semiconductor materials that could replace the TiO_2_ for the perovskite-based nanostructured solar cells are needed. In addition to the preparation method, the electronic energy levels (EELs) of those substitute materials are needed to match the EELs of the perovskite materials for efficient charge transfer. For this purpose, ZnO compact layer and ZnO nanorods are recently prepared by electrodeposition and chemical bath deposition, respectively, to replace the TiO_2_ by Kumar et al. [25]. It is worth noting that the materials used to replace the TiO_2_ do not necessarily have to be the n-type semiconductors such as the ZnO because the perovskites can conduct not only positive holes [22,26] but also electrons [16].

**Figure 1:**
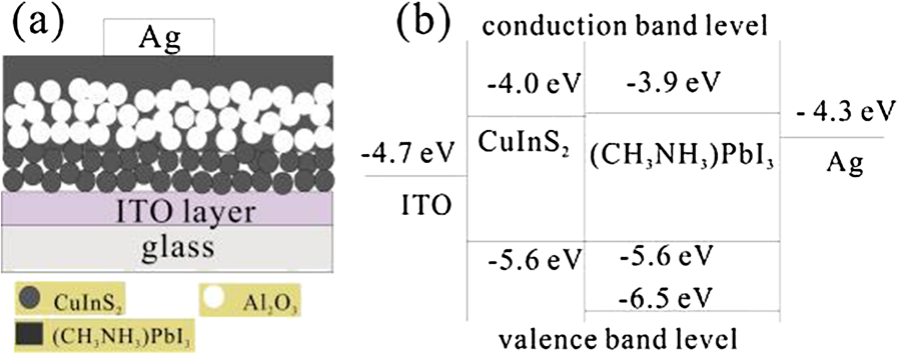
**Schematic diagram (a) and energy diagram (b) of the ITO/CuInS**_**2**_**/Al**_**2**_**O**_**3**_**/(CH**_**3**_**NH**_**3**_**)PbI**_**3**_**/Ag device.**

It is known that CuInS_2_ as a p-type semiconductor is a very promising light-absorbing material for its direct bandgap of 1.5 eV, which is closely matched to the best bandgap (1.45 eV) of the solar cell materials [27]. Recently, a method of using a molecular-based precursor solution to synthesize CuInS_2_ nanocrystals at relatively lower temperatures (250°C) has been presented by Li et al. [10]. More importantly, the valence band level (−5.6 eV) of the CuInS_2_ is also matched to that (−5.6 or −6.5 eV) [13] of the (CH_3_NH_3_)PbI_3_, which is very beneficial to the hole transfer from the (CH_3_NH_3_)PbI_3_ to the CuInS_2_. Therefore, replacing the TiO_2_ with CuInS_2_ is reasonable. In this study, for the first time, the p-type semiconductor material, CuInS_2_, as both the light harvester and hole transporter is prepared by the reported method [10] to replace the commonly used n-type TiO_2_ in the perovskite-based solar cells. Moreover, the indium tin oxide (ITO) glass rather than the commonly used fluorine-doped tin oxide (FTO) glass in previously reported perovskite-based solar cells is used as a light-incidence electrode because the CuInS_2_ film can directly be deposited on the ITO glass at a temperature as low as 250°C. After the deposition of CuInS_2_ film, the Al_2_O_3_ and (CH_3_NH_3_)PbI_3_ are successively deposited on the CuInS_2_ film to form the CuInS_2_/(CH_3_NH_3_)PbI_3_ planar heterojunction. The porous Al_2_O_3_ layer acts as a scaffold. Finally, an evaporated Ag top electrode was deposited on the (CH_3_NH_3_)PbI_3_ at a pressure of 10^−6^ Torr to complete the device fabrication. The schematics and energy diagram of the prepared solar cells are shown in Figure 1a, b, respectively. The surface morphology, structure characterization, and optical property of the prepared CuInS_2_/(CH_3_NH_3_)PbI_3_ film are studied. Furthermore, the influence of the thickness of the CuInS_2_ film on the power conversion efficiency of the fabricated CuInS_2_/(CH_3_NH_3_)PbI_3_ planar heterojunction solar cell is investigated.

## Methods

### Materials

Indium acetate (In(OAc)_3_, 99.99%), copper iodode (CuI, 99.999%), thiourea (≥99.0%), 1-propionic acid (≥99.5%), γ-butyrolactone (≥99.0%), aluminum oxide (Al_2_O_3_, 20 wt.% in isopropanol), methylamine (40 wt.% in H_2_O), hydroiodic acid (57 wt.% in water), diethyl ether, and PbI_2_ (99.999%) were purchased from Sigma-Aldrich (St. Louis, MO, USA). All the reagents were used without further purification. Indium tin oxide-coated glass slides (ITO, ≤15 Ω/square, Wuhu Token Sci. Co., Ltd., China) were cleaned by successive ultrasonic treatment in deionized water, acetone, and isopropyl alcohol and then dried at 100°C for 10 min.

### Synthesis of CuInS_2_ nanocrystal film on ITO substrate

CuInS_2_ nanocrystals were synthesized through a spin-coating method, which is similar to that reported by Li et al. [10]. Briefly, CuI (0.11 mmol), In(OAc)_3_ (0.1 mmol), and thiourea (0.5 mmol) were dissolved in a mixture of 1-butylamine (0.6 mL) and 1-propionic acid (40 μL) under a nitrogen atmosphere in a glovebox (O_2_ < 0.1 ppm, H_2_O < 0.1 ppm). The mixture was shaken for 1 min, and after which, the obtained CuInS_2_ precursor solution was then spin-cast onto the cleaned ITO substrates at 4,000 rpm for 30 s. Then, the obtained films were calcined at 150°C for 10 min and then heated to 250°C and held for 15 min at this temperature. To change the thickness of the CuInS_2_ film, a spinning-drying cycle was repeated several times. The ITO/CuInS_2_ sample after *n* cycles of CuInS_2_ deposition was denoted as ITO/CuInS_2_(*n*).

### Synthesis of methylammonium iodide and (CH_3_NH_3_)PbI_3_

Methylammonium iodide (CH_3_NH_3_I) was synthesized by reacting methylamine (aqueous, 40 wt.%) and hydroiodic acid (aqueous, 57 wt.%) in an ice bath for 2 h with stirring, as described elsewhere [18]. After that, the solvent was evaporated and the precipitate was washed using diethyl ether three times and dried at 60°C for 24 h in a vacuum oven. The resulting product, CH_3_NH_3_I, was used without further purification. To obtain a (CH_3_NH_3_)PbI_3_ precursor, the synthesized CH_3_NH_3_I was mixed with PbI_2_ at a 1:1 mol ratio in γ-butyrolactone (40% by weight) at 60°C.

### Solar cell fabrication

First, a Al_2_O_3_ layer was introduced on the ITO/CuInS_2_(*n*) films by spin coating isopropanol solution containing Al_2_O_3_ nanoparticles at 4,000 rpm for 60 s. After that, the films were dried at 150°C for 30 min to obtain ITO/CuInS_2_(*n*)/Al_2_O_3_. Then, the prepared ITO/CuInS_2_(*n*)/Al_2_O_3_ films were spin-coated with the obtained (CH_3_NH_3_)PbI_3_/γ-butyrolactone solution at 3,000 rpm for 60 s and then dried at 100°C for 1 h to form crystalline (CH_3_NH_3_)PbI_3_. The prepared ITO/CuInS_2_(*n*)/Al_2_O_3_/(CH_3_NH_3_)PbI_3_ films were naturally cooled to room temperature. All the experiment was finished in a nitrogen glovebox (O_2_ < 0.1 ppm, H_2_O < 0.1 ppm). Finally, a silver back contact layer was deposited by thermal evaporation onto the ITO/CuInS_2_(*n*)/(CH_3_NH_3_)PbI_3_ films from a silver wire (99.999%).

### Characterization

The surface morphology and structure of the prepared ITO/CuInS_2_(*n*) and ITO/CuInS_2_(*n*)/Al_2_O_3_/(CH_3_NH_3_)PbI_3_ films were characterized using a scanning electron microscope (SEM) (JSM-7001 F, Japan Electron Optics Laboratory Co., Ltd., Tokyo, Japan) and power X-ray diffractometry (XRD) (DX-2500, Dandong Fangyuan Instrument Co., Ltd., Dandong, China), respectively. It should be noted that, for XRD measurement, the CuInS_2_ and (CH_3_NH_3_)PbI_3_ films are individually deposited on the cleaned glass without ITO layer to exclude the influence of the substrate on the XRD measurement. UV-visible absorption measurements were conducted using a UV–vis spectrophotometer (UV-2550, Shimadzu Corporation, Kyoto, Japan). Current density-voltage (*J*-*V*) characteristics of the as-prepared solar cells were measured using a Keithley 2410 SourceMeter (Keithley Instruments, Inc., Cleveland, OH, USA). A solar simulator (Newport Inc., Irvine, CA, USA) was used as the light source to provide AM 1.5 G simulated solar light (100 mW/cm^2^). Before each measurement, the light intensity was determined using a calibrated Si reference diode. For all measurements, the effective illumination area of the cells was 4 mm^2^. The monochromatic incident photon-to-electron conversion efficiency (IPCE) spectra for the fabricated solar cells were measured using a commercial setup (QTest Station 2000 IPCE Measurement System, Crowntech, Macungie, PA, USA).

## Results and discussion

**Figure 2:**
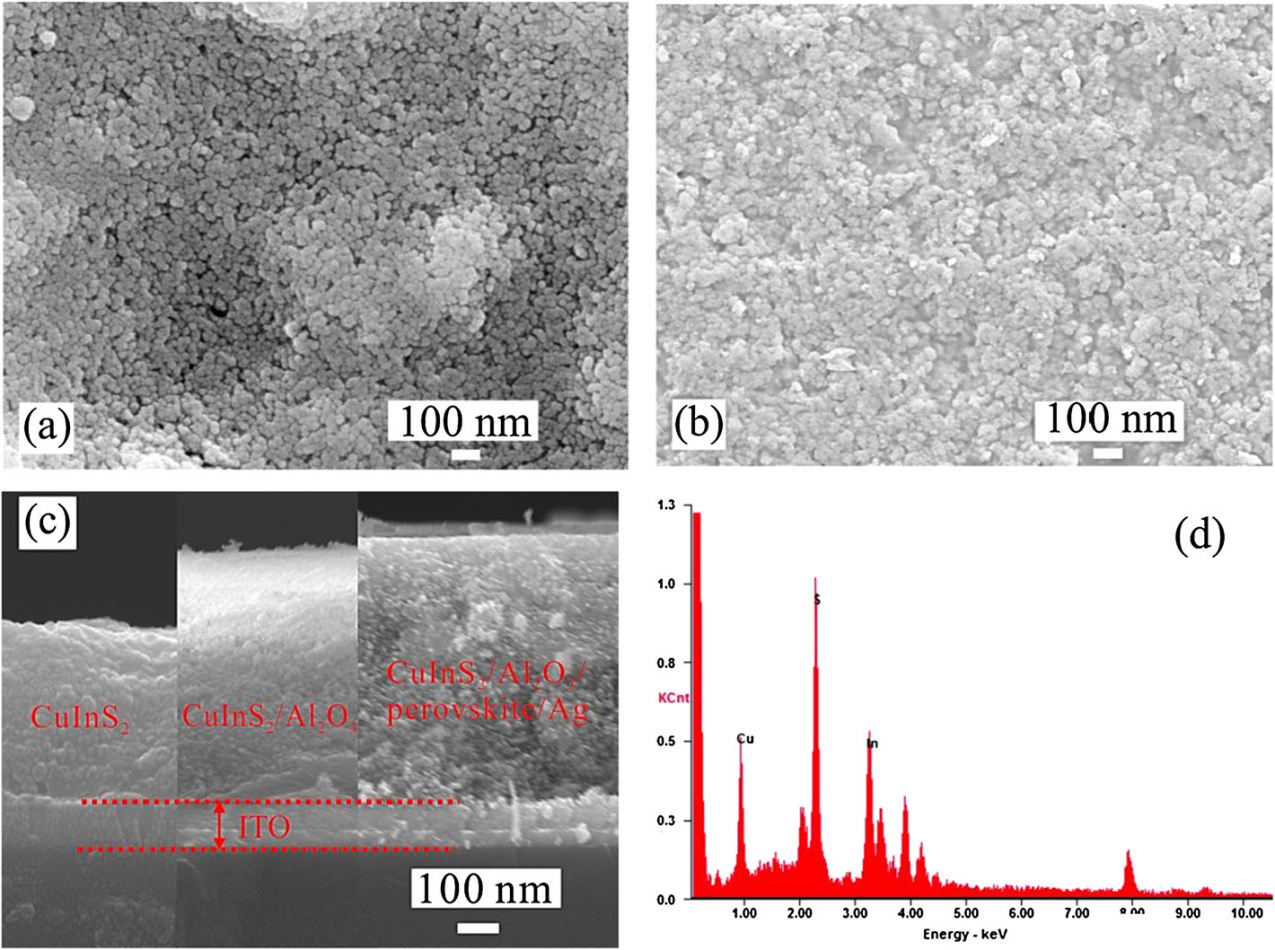
**The morphology, structures, and chemical composition of the as-prepared CuInS**_**2**_**. (a)** Top view of the ITO/CuInS_2_(1) film; **(b)** the top-view SEM image of the ITO/CuInS_2_(2) film; **(c)** the cross-sectional images of the ITO/CuInS_2_(2), ITO/CuInS_2_(2)/Al_2_O_3_, and ITO/CuInS_2_(2)/Al_2_O_3_/(CH_3_NH_3_)PbI_3_/Ag films; and **(d)** the EDX spectrum of the ITO/CuInS_2_(2) film.

The morphology, structures, and chemical composition of the as-prepared CuInS_2_ were studied with SEM studies accompanied by energy dispersive X-ray spectrometry (EDX). Figure 2a shows a typical top-view SEM image of the ITO/CuInS_2_(1) film. As shown in Figure 2a, the surface of the ITO substrate is covered with the CuInS_2_ film. The CuInS_2_ film is composed of CuInS_2_ nanoparticles, and these CuInS_2_ nanoparticles appear to be fused together after heating at 250°C. Moreover, some voids can be clearly seen in the ITO/CuInS_2_ (1) film, which can be explained by the decomposition of volatile surface ligands and precurse materials [10]. For a comparison, the SEM top image of the ITO/CuInS_2_(2) is displayed in Figure 2b. It can be observed that, compared to the ITO/CuInS_2_(1) film, the number of the voids in the ITO/CuInS_2_(2) film decreases significantly, indicating that the voids in the ITO/CuInS_2_(1) have been filled by the CuInS_2_ precursor after two times of spin coating. In addition, the cross-sectional SEM images of the ITO/CuInS_2_(2), ITO/CuInS_2_(2)/Al_2_O_3_, and ITO/CuInS_2_(2)/Al_2_O_3_/(CH_3_NH_3_)PbI_3_/Ag films are shown in Figure 2c. It clearly shows that the CuInS_2_(2) film with an average thickness of 400 nm is formed on the ITO glass, and there are no obvious voids in the film. After the deposition of Al_2_O_3_, the thickness of the ITO/CuInS_2_(2)/Al_2_O_3_ film increased. After the deposition of (CH_3_NH_3_) PbI_3_, the thickness of the ITO/CuInS_2_ (2)/Al_2_O_3_/(CH_3_NH_3_) PbI_3_ film further increased to about 650 nm. The more important thing is that the (CH_3_NH_3_) PbI_3_ precursor solution permeated into the porous Al_2_O_3_ layer to form the (CH_3_NH_3_) PbI_3_. The corresponding EDX spectrum of ITO/CuInS_2_ (2) is shown in Figure 2d, which shows the film is mainly composed of copper (Cu), indium (In), and sulfur (S). The chemical compositional analysis reveals that the atomic ratio of Cu, In, and S is 25.33%, 24.9%, and 49.77%, respectively, close to 1:1:2, which confirms the formation of CuInS_2_.

**Figure 3:**
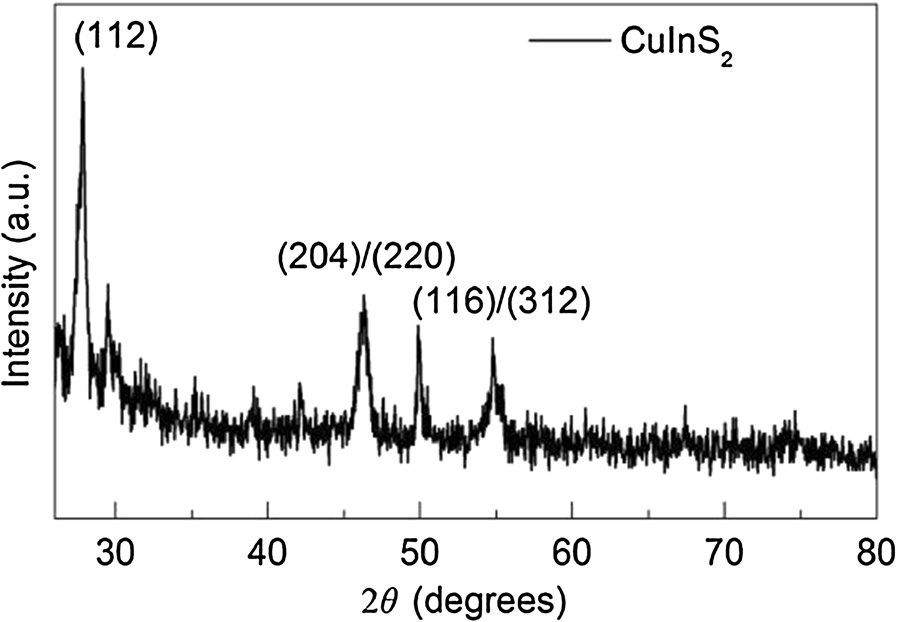
**The XRD pattern of the as-prepared CuInS**_**2**_**(3) film.**

To characterize the crystal structure of the CuInS_2_, a typical XRD pattern of the as-prepared CuInS_2_(3) film on a clean glass substrate is shown in Figure 3. The well-defined peaks can be referred to a tetragonal CuInS_2_ (112), (204), (220), (116), and (312) (JCPDS file no. 85–1575), which is in agreement with the reported results [10,28,29].

**Figure 4:**
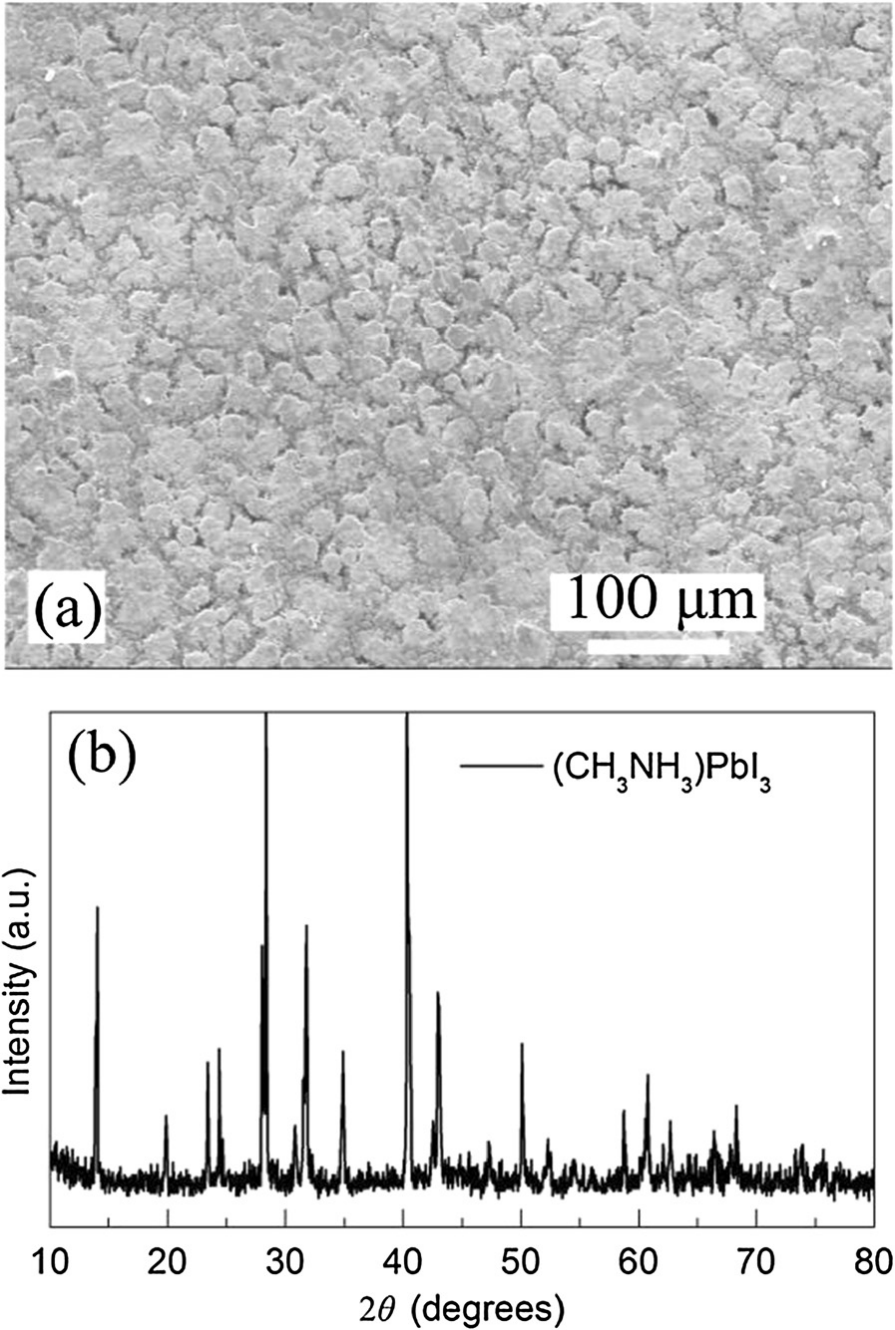
**SEM image and XRD analysis. (a)** Top-view SEM image of the ITO/CuInS_2_(2)/Al_2_O_3_/(CH_3_NH_3_)PbI_3_ film and **(b)** the XRD analysis of the prepared (CH_3_NH_3_)PbI_3_ film.

Figure 4a shows a top-view SEM image of the ITO/CuInS_2_(2)/Al_2_O_3_/(CH_3_NH_3_)PbI_3_ film. By comparing this image with that (Figure 2b) of the ITO/CuInS_2_(2) film, it can be clearly observed that the (CH_3_NH_3_)PbI_3_ film was deposited on the CuInS_2_. However, the solution-processed (CH_3_NH_3_)PbI_3_ films are not very uniform and coated the CuInS_2_ film only partially with micrometer-sized (CH_3_NH_3_)PbI_3_ platelets, which is very similar to the observed phenomenon in the (CH_3_NH_3_)PbI_3_-coved compact TiO_2_ film [21]. Furthermore, to characterize the crystal structure and phase composition of the synthesized (CH_3_NH_3_)PbI_3_ film, the XRD analysis of the prepared (CH_3_NH_3_)PbI_3_ film was performed and shown in Figure 4b. It can be seen from Figure 4b that the diffraction peaks are in good agreement with the tetragonal phase of the (CH_3_NH_3_) PbI_3_ perovskite [17,30].

**Figure 5:**
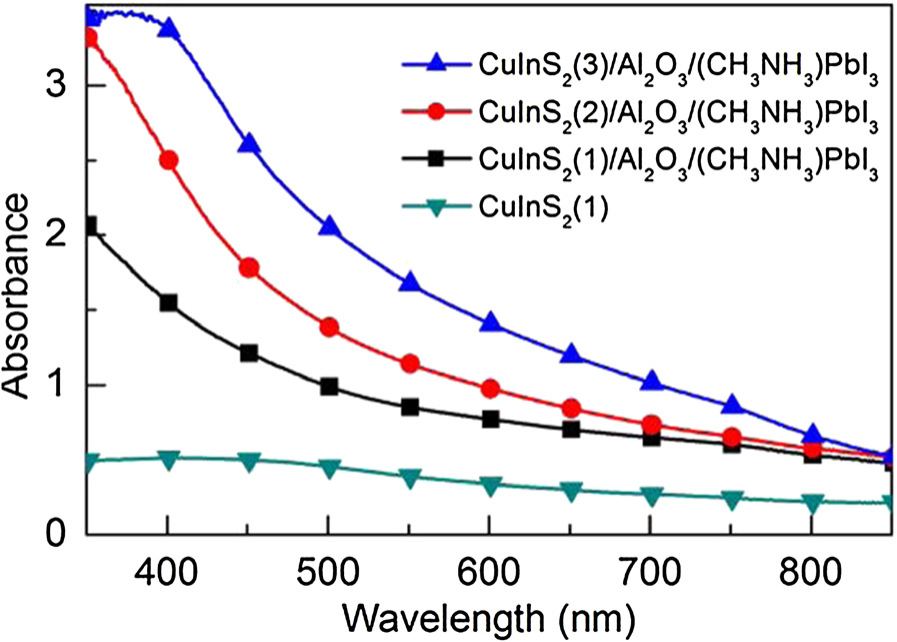
**UV–vis absorption spectra of the ITO/CuInS**_**2**_**(1) and ITO/CuInS**_**2**_**( *n *)/Al**_**2**_**O**_**3**_**/(CH**_**3**_**NH**_**3**_**)PbI**_**3 **_**films (*n =*** **1, 2, and 3).**

To study the light absorption properties of the prepared ITO/CuInS_2_(*n*) and ITO/CuInS_2_(*n*)/Al_2_O_3_/(CH_3_NH_3_)PbI_3_ films for application in photovoltaic devices, light absorption studies are carried out. Figure 5 shows the UV–vis absorption spectra of the ITO/CuInS_2_(1) and ITO/CuInS_2_(*n*)/Al_2_O_3_/(CH_3_NH_3_)PbI_3_ films (*n =* 1, 2, and 3). As shown in Figure 5, the ITO/CuInS_2_(1) film has light absorption at wavelengths below 825 nm, which is similar to the reported results [10,31,32]. After the (CH_3_NH_3_)PbI_3_ film was deposited on the ITO/CuInS_2_(1) film, the absorbance of the spectra of the ITO/CuInS_2_(1)/Al_2_O_3_/(CH_3_NH_3_)PbI_3_ film increases significantly in the UV region as well as the visible region. For the ITO/CuInS_2_(*n*)/Al_2_O_3_/(CH_3_NH_3_)PbI_3_ films after other spin-cast cycles (*n =* 2 and 3) in our experiments, similar results are also obtained, which can be attributed to the light absorption of the deposited (CH_3_NH_3_)PbI_3_ film. Moreover, for the ITO/CuInS_2_(*n*)/Al_2_O_3_/(CH_3_NH_3_)PbI_3_ films, Figure 5 also illustrates that the light absorbance was enhanced with an increase in spin-cast cycle number *n*, indicating an increased deposition amount of CuInS_2_. In addition, the absorption lines of the ITO/CuInS_2_/(CH_3_NH_3_)PbI_3_ films are very similar to those of the reported FTO/TiO_2_/(CH_3_NH_3_)PbI_3_ films [33], which further confirms the formation of (CH_3_NH_3_)PbI_3_ film and shows the potential of the ITO/CuInS_2_/Al_2_O_3_/(CH_3_NH_3_)PbI_3_ films in photovoltaic application.

**Figure 6:**
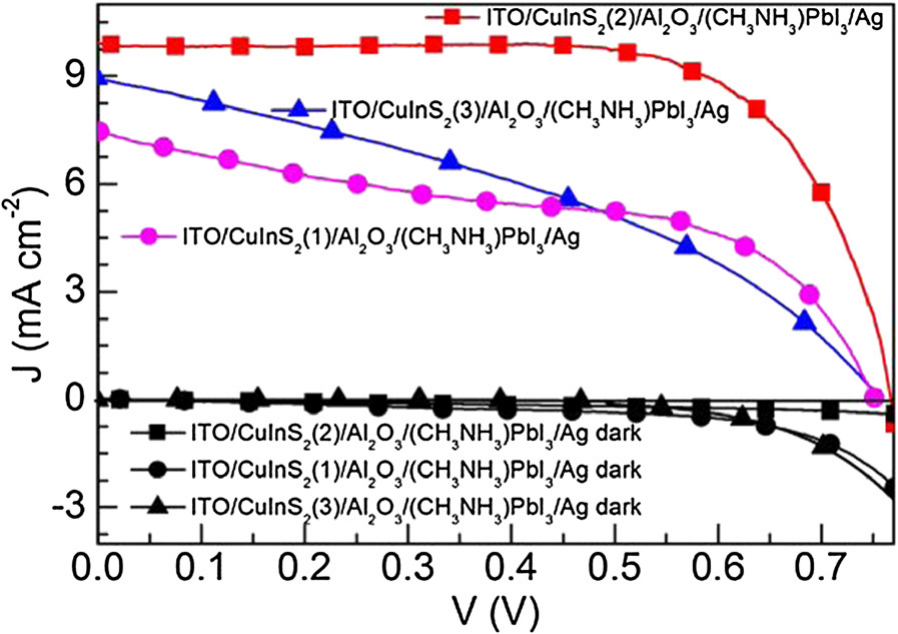
***J*-*V *characteristics.***J*-*V* characteristics of the ITO/CuInS_2_(*n*)/Al_2_O_3_/(CH_3_NH_3_)PbI_3_/Ag solar cells under simulated AM 1.5G solar irradiation and in the dark (*n* = 1, 2, and 3).

The *J*-*V* characteristics of the ITO/CuInS_2_(*n*)/Al_2_O_3_/(CH_3_NH_3_)PbI_3_/Ag solar cells under simulated AM 1.5 G solar irradiation and in the dark are shown in Figure 6. All device parameters under the light illumination, the open-circuit voltage (*V*_oc_), the short-circuit photocurrent (*J*_sc_), the fill factor (FF), and the solar power conversion efficiency (*η*), extracted from the *J*-*V* characteristics are summarized in Table 1. For the ITO/CuInS_2_(*n*)/Al_2_O_3_/(CH_3_NH_3_)PbI_3_/Ag cells, with the increase of CuInS_2_ deposition number *n* from 1 to 2, the *J*_sc_ increased from 8.85 to 9.92 mA/cm^2^, the *V*_oc_ increased from 0.74 to 0.76 V, the FF increased from 0.51 to 0.70, and the *η* increased from 3.31% to 5.30%. These results might be caused by the voids in the CuInS_2_ film. As shown in Figure 2a, some voids have been found in the ITO/CuInS_2_(1) film. When the (CH_3_NH_3_)PbI_3_ precursor solution was spin-cast onto the ITO/CuInS_2_(1)/Al_2_O_3_ film, these voids might be filled by the (CH_3_NH_3_)PbI_3_ precursor solution. Therefore, similar to the observed phenomenon in the mesoporous-TiO_2_/(CH_3_NH_3_)PbI_3_ film, the (CH_3_NH_3_)PbI_3_ probably infiltrated to the bottom of the CuInS_2_ film and had a contact with the hole collection electrode (i.e., the ITO electrode), which will enhance the probability of recombination between electrons in the (CH_3_NH_3_)PbI_3_ and holes in the ITO in the ITO/CuInS_2_(1)/Al_2_O_3_/(CH_3_NH_3_)PbI_3_/Ag solar cell. In contrast, as shown in Figure 2c, there are few voids in the ITO/CuInS_2_(2) film, which may effectively reduce the charge recombination at the ITO/(CH_3_NH_3_)PbI_3_ interface in the ITO/CuInS_2_(2)/Al_2_O_3_/(CH_3_NH_3_)PbI_3_/Ag solar cell. This explanation is supported by the *J*-*V* characteristics of the ITO/CuInS_2_(*n*)/(CH_3_NH_3_)PbI_3_/Ag in the dark (Figure 6) since the charge recombination can be typically represented by the dark current [2,34,35]. It can be observed that the dark current density of the ITO/CuInS_2_(2)/Al_2_O_3_/(CH_3_NH_3_)PbI_3_/Ag cell is lower than that of the ITO/CuInS_2_(1)/Al_2_O_3_/(CH_3_NH_3_)PbI_3_/Ag cell, which indicates that the charge recombination is reduced in the ITO/CuInS_2_(2)/Al_2_O_3_/(CH_3_NH_3_)PbI_3_/Ag cell.

**Table 1:** **Summary of device performance under white light illumination with an intensity of 100 mW/cm**^**2**^

**Cells**	***V***_***oc ***_**(V)**	***J***_**sc **_**(mA/cm**^**−2**^**)**	**FF**	**PCE (%)**
ITO/CuInS_2_(1)/Al_2_O_3_/(CH_3_NH_3_)PbI_3_/Ag	0.74	8.85	0.51	3.31
ITO/CuInS_2_(2)/Al_2_O_3_/(CH_3_NH_3_)PbI_3_/Ag	0.76	9.92	0.70	5.30
ITO/CuInS_2_(3)/Al_2_O_3_/(CH_3_NH_3_)PbI_3_/Ag	0.76	8.98	0.38	2.60

However, the device performance of the ITO/CuInS_2_ (*n*)/Al_2_O_3_/(CH_3_NH_3_)PbI_3_/Ag cell decreased as the CuInS_2_ deposition number *n* increased further from 2 to 3. It can be found from Table 1 that, compared to the ITO/CuInS_2_ (2)/Al_2_O_3_/(CH_3_NH_3_) PbI_3_/Ag cell, all device parameters (*J*_sc_, FF, and *η*) except *V*_oc_ of the ITO/CuInS_2_(3)/Al_2_O_3_/(CH_3_NH_3_) PbI_3_/Ag cell decreased. For the ITO/CuInS_2_ (3)/Al_2_O_3_/(CH_3_NH_3_)PbI_3_/Ag cell, the *J*_sc_, FF, and *η* decreased to 8.98 mA/cm^2^, 0.38, and 2.60%, respectively. The main reason for the decreased device performance may be the increased thickness of CuInS_2_ film. As shown in Figure 1, in the ITO/CuInS_2_/Al_2_O_3_/(CH_3_NH_3_)PbI_3_/Ag cell, the CuInS_2_ mainly conducted holes. Therefore, increasing the thickness of CuInS_2_ film would increase the hole-transfer resistance and lead to an increase in overall series resistance (*R*_s_) in the cells, which would inevitably lead to the degradation of *J*_sc_ and FF. The *R*_s_ can be calculated from the inverse slope of the illuminated *J*-*V* characteristics at *J =* 0. The *R*_s_ values for the ITO/CuInS_2_(2)/Al_2_O_3_/(CH_3_NH_3_)PbI_3_/Ag and ITO/CuInS_2_(3)/Al_2_O_3_/(CH_3_NH_3_)PbI_3_/Ag cells are 6.4 and 29.6 Ω/cm^2^, respectively, which were calculated from the illuminated *J*-*V* characteristics (shown in Figure 6). Obviously, compared to the ITO/CuInS_2_(2)/Al_2_O_3_/(CH_3_NH_3_)PbI_3_/Ag cell, the *R*_s_ of ITO/CuInS_2_(3)/Al_2_O_3_/(CH_3_NH_3_)PbI_3_/Ag cell increased due to the increased thickness of the CuInS_2_ film. Furthermore, a too thick CuInS_2_ film may dramatically reduce the amount of light absorbed by the (CH_3_NH_3_)PbI_3_ film, which results in a sizeable reduction in the number of the photo-generated electrons in the (CH_3_NH_3_)PbI_3_ film and therefore reduces the *J*_sc_ and FF.

**Figure 7:**
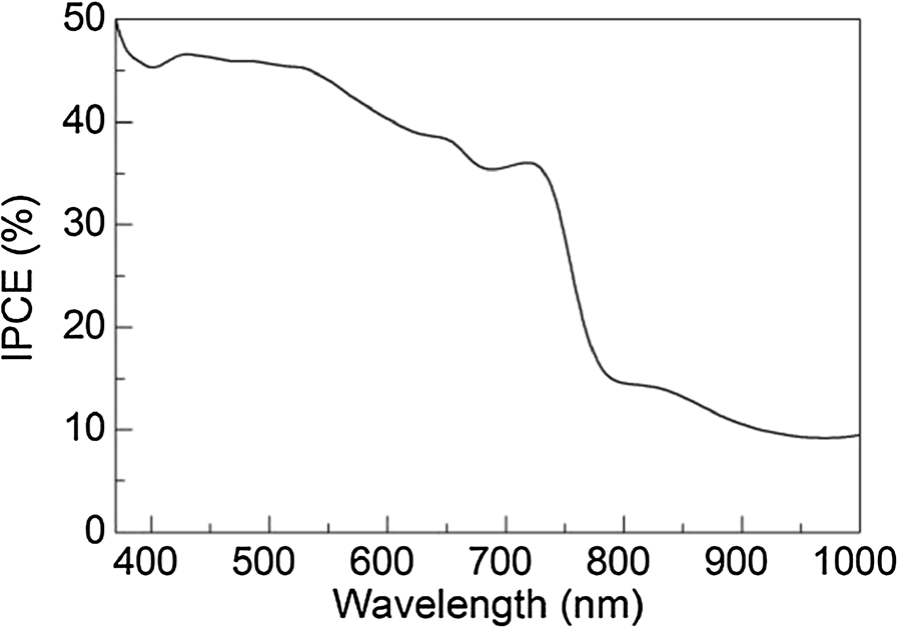
**IPCE spectra of the ITO/CuInS**_**2**_**( *2 *)/Al**_**2**_**O**_**3**_**/(CH**_**3**_**NH**_**3**_**)PbI**_**3**_**/Ag solar cell.**

Figure 7 shows the IPCE spectra of the ITO/CuInS_2_(*2*)/Al_2_O_3_/(CH_3_NH_3_)PbI_3_/Ag solar cell. It can be observed that the solar cell shows a spectral response in the almost entire wavelength region from 370 to 1,000 nm. The IPCE of over 31% is observed at a wavelength range from 370 to 750 nm. Furthermore, for the IPCE value, a sharp decrease in the wavelength region from 750 to 820 nm is observed. The threshold wavelength of 820 nm is related to the bandgap of about 1.5 eV for (CH_3_NH_3_)PbI_3_[36]. These results are in agreement with the previously reported IPCE spectra for the perovskite solar cells without a CuInS_2_ layer [19,36,37]. It should be noted that, for these reported perovskite solar cells, the IPCE values are nearly zero in the long wavelength region (820 to 1,000 nm). However, in our case, IPCE of over 9% is observed at an entire wavelength range from 820 to 1,000 nm, resulting from the photocurrent originating from the CuInS_2_ layer. Therefore, the CuInS_2_ layer can improve the IPCE values of the solar cells in the long wavelength region.

Our experimental results demonstrated that, for the ITO/CuInS_2_(*n*)/Al_2_O_3_/(CH_3_NH_3_)PbI_3_/Ag cells, the ITO/CuInS_2_(2)/Al_2_O_3_/(CH_3_NH_3_)PbI_3_/Ag cell showed the highest solar power conversion efficiency of 5.30%. However, it should be noted that the highest power conversion efficiency presented here was just taken from the solar cells with a simplified architecture. The solar cell architecture can be further optimized. For example, a hole-selective layer can be inserted between the ITO and the CuInS_2_ layers to reduce the charge recombination at the ITO/CuInS_2_ interface. Similarly, inserting an electron-selective layer between the (CH_3_NH_3_)PbI_3_ layer and the Ag electrode may also suppress the charge recombination at the (CH_3_NH_3_)PbI_3_/Ag interface. Therefore, the solar cells with an architecture that incorporates the charge (hole or electron)-selective layer may achieve higher power conversion efficiency, which is our future study.

## Conclusions

In summary, the solution-processed (CH_3_NH_3_)PbI_3_ perovskite/CuInS_2_ planar heterojunction solar cells with a Al_2_O_3_ scaffold have been successfully fabricated, in which the CuInS_2_ films as both the light harvester and hole transporter were prepared at a relatively low temperature (250°C) via a simple solution-based chemical approach to replace the commonly used n-type TiO_2_ layer. The influence of the thickness of CuInS_2_ film on the performance of the fabricated ITO/CuInS_2_/Al_2_O_3_/(CH_3_NH_3_)PbI_3_/Ag solar cells was investigated. Our experimental results demonstrated that an optimum power conversion efficiency of up to 5.30% can be achieved by the ITO/CuInS_2_(2)/Al_2_O_3_/(CH_3_NH_3_)PbI_3_/Ag cell. Optimizing the device architecture may further improve the performance of the ITO/CuInS_2_(*n*)/Al_2_O_3_/(CH_3_NH_3_)PbI_3_/Ag solar cells. The present research findings offer a new approach to achieve low-cost and high-efficiency solar cells.

## Competing interests

The authors declare that they have no competing interests.

## Authors’ contributions

CC carried out the experiments, participated in the sequence alignment, and drafted the manuscript. FL participated in the device preparation. FW and FT participated in the design of the study. CL and YZ performed the statistical analysis. WZ conceived of the study and helped to draft the manuscript. All authors read and approved the final manuscript.

## References

[B1] Suryawanshi MP Agawane GL Bhosale SM Shin SW Patil PS Kim JH Moholkar AV CZTS based thin film solar cells: a status review Mater Technol 2013 28 98 109 10.1179/1753555712Y.0000000038

[B2] Barkhouse DAR Gunawan O Gokmen T Todorov TK Mitzi DB Device characteristics of a 10.1% hydrazine-processed Cu2ZnSn(Se, S)4 solar cell Prog Photovolt Res Appl 2012 20 6 11 10.1002/pip.1160

[B3] Greätzel M Janssen RAJ Mitzi DB Sargent EH Materials interface engineering for solution-processed photovoltaics Nature 2012 488 304 312 10.1038/nature11476 22895335

[B4] Chang LY Lunt RR Brown PR Bulovic V Bawendi MG Low-temperature solution-processed solar cells based on PbS colloidal quantum Dot/CdS heterojunctions Nano Lett 2013 13 994 999 10.1021/nl3041417 23406331

[B5] Rath AK Bernechea M Martinez L de Arquer FPG Osmond J Konstantatos G Solution-processed inorganic bulk nano-heterojunctions and their application to solar cells Nat Photonics 2012 6 529 534 10.1038/nphoton.2012.139

[B6] Tian YY Zhang YJ Lin YZ Gao K Zhang YP Liu KY Yang QQ Zhou X Qin DH Wu HB Xia YX Hou LT Lan LF Chen JW Wang D Yao RH Solution-processed efficient CdTe nanocrystal/CBD-CdS hetero-junction solar cells with ZnO interlayer J Nanopart Res 2013 15 2053

[B7] Gur I Fromer NA Geier ML Alivisatos AP Air-stable all-inorganic nanocrystal solar cells processed from solution Science 2005 310 462 465 10.1126/science.1117908 16239470

[B8] Gur I Fromer NA Chen CP Kanaras AG Alivisatos AP Hybrid solar cells with prescribed nanoscale morphologies based on hyperbranched semiconductor nanocrystals Nano Lett 2007 7 409 414 10.1021/nl062660t 17298008

[B9] Azimi H Heumuller T Gerl A Matt G Kubis P Distaso M Ahmad R Akdas T Richter M Peukert W Brabec CJ Relation of nanostructure and recombination dynamics in a Low-temperature solution-processed CuInS_2_ nanocrystalline solar cell Adv Energ Mater 2013 3 1589 1596 10.1002/aenm.201300449

[B10] Li L Coates N Moses D Solution-processed inorganic solar cell based on in situ synthesis and film deposition of CuInS_2_ nanocrystals J Am Chem Soc 2010 132 22 23 10.1021/ja908371f 20000465

[B11] Todorov T Sugimoto H Gunawan O Gokmen T Mitzi DB High-efficiency devices with pure solution-processed Cu2ZnSn(S, Se) (4) absorbers IEEE J Photovolt 2014 4 483 485

[B12] Hodes G Perovskite-based solar cells Science 2013 342 317 318 10.1126/science.1245473 24136955

[B13] Xing GC Mathews N Sun SY Lim SS Lam YM Grätzel M Mhaisalkar S Sum TC Long-range balanced electron- and hole-transport lengths in organic–inorganic CH_3_NH_3_PbI_3_ Science 2013 342 344 347 10.1126/science.1243167 24136965

[B14] Stranks SD Eperon GE Grancini G Menelaou C Alcocer MJP Leijtens T Herz LM Petrozza A Snaith HJ Electron–hole diffusion lengths exceeding 1 micrometer in an organometal trihalide perovskite absorber Science 2013 342 341 344 10.1126/science.1243982 24136964

[B15] Kim HS Lee CR Im JH Lee KB Moehl T Marchioro A Moon SJ Humphry-Baker R Yum JH Moser JE Grätzel M Park NG Lead iodide perovskite sensitized all-solid-state submicron thin film mesoscopic solar cell with efficiency exceeding 9% Sci Rep UK 2012 2 591 10.1038/srep00591 PMC3423636 22912919

[B16] Lee MM Teuscher J Miyasaka T Murakami TN Snaith HJ Efficient hybrid solar cells based on meso-superstructured organometal halide perovskites Science 2012 338 643 647 10.1126/science.1228604 23042296

[B17] Burschka J Pellet N Moon S-J Humphry-Baker R Gao P Nazeeruddin MK Gräetzel M Sequential deposition as a route to high-performance perovskite-sensitized solar cells Nature 2013 499 316 319 10.1038/nature12340 23842493

[B18] Heo JH Im SH Noh JH Mandal TN Lim CS Chang JA Lee YH Kim HJ Sarkar A Nazeeruddin MK Grätzel M Seok SI Efficient inorganic–organic hybrid heterojunction solar cells containing perovskite compound and polymeric hole conductors Nat Photonics 2013 7 487 492

[B19] Abrusci A Stranks SD Docampo P Yip HL Jen AKY Snaith HJ High-performance perovskite-polymer hybrid solar cells via electronic coupling with fullerene monolayers Nano Lett 2013 13 3124 3128 10.1021/nl401044q 23772773

[B20] Park NG Organometal perovskite light absorbers toward a 20% efficiency low-cost solid-state mesoscopic solar cell J Phys Chem Lett 2013 4 2423 2429 10.1021/jz400892a

[B21] Liu MZ Johnston MB Snaith HJ Efficient planar heterojunction perovskite solar cells by vapour deposition Nature 2013 501 395 398 10.1038/nature12509 24025775

[B22] Etgar L Gao P Xue ZS Peng Q Chandiran AK Liu B Nazeeruddin MK Grätzel M Mesoscopic CH_3_NH_3_PbI_3_/TiO_2_ heterojunction solar cells J Am Chem Soc 2012 134 17396 17399 10.1021/ja307789s 23043296

[B23] Ball JM Lee MM Hey A Snaith HJ Low-temperature processed meso-superstructured to thin-film perovskite solar cells Energ Environ Sci 2013 6 1739 1743 10.1039/c3ee40810h

[B24] Carnie MJ Charbonneau C Davies ML Troughton J Watson TM Wojciechowski K Snaith H Worsley DA A one-step low temperature processing route for organolead halide perovskite solar cells Chem Commun 2013 49 7893 7895 10.1039/c3cc44177f 23900427

[B25] Kumar MH Yantara N Dharani S Gräetzel M Mhaisalkar S Boix PP Mathews N Flexible, low-temperature, solution processed ZnO-based perovskite solid state solar cells Chem Commun (Camb) 2013 49 11089 11091 10.1039/c3cc46534a 24141601

[B26] Chung I Lee B He JQ Chang RPH Kanatzidis MG All-solid-state dye-sensitized solar cells with high efficiency Nature 2012 485 U486 U494 10.1038/nature11067 22622574

[B27] Tell B Shay JL Kasper HM Electrical properties, optical properties, and band structure of CuGaS_2_ and CuInS_2_ Phys Rev Biol 1971 4 2463 2471 10.1103/PhysRevB.4.2463

[B28] Li TL Teng HS Solution synthesis of high-quality CuInS_2_ quantum dots as sensitizers for TiO_2_ photoelectrodes J Mater Chem 2010 20 3656 3664 10.1039/b927279h

[B29] Shi L Yin PQ Wang LB Qian YT Fabrication of single-crystalline CuInS_2_ nanowires array via a diethylenetriamine-thermal route Crystengcomm 2012 14 7217 7221 10.1039/c2ce25368b

[B30] Baikie T Fang YN Kadro JM Schreyer M Wei FX Mhaisalkar SG Gräetzel M White TJ Synthesis and crystal chemistry of the hybrid perovskite (CH_3_NH_3_) PbI_3_ for solid-state sensitised solar cell applications J Mater Chem A 2013 1 5628 5641

[B31] Zhou ZJ Fan JQ Wang X Sun WZ Zhou WH Du ZL Wu SX Solution fabrication and photoelectrical properties of CuInS_2_ nanocrystals on TiO_2_ nanorod array Acs Appl Mater Interfaces 2011 3 2189 2194 10.1021/am200500k 21688822

[B32] Chen C Ali G Yoo SH Kum JM Cho SO Improved conversion efficiency of CdS quantum dot-sensitized TiO_2_ nanotube-arrays using CuInS_2_ as a co-sensitizer and an energy barrier layer J Mater Chem 2011 21 16430 16435 10.1039/c1jm13616j

[B33] Im J-H Lee C-R Lee J-W Park S-W Park N-G 6.5% efficient perovskite quantum-dot-sensitized solar cell Nanoscale 2011 3 4088 4093 10.1039/c1nr10867k 21897986

[B34] Na SI Kim TS Oh SH Kim J Kim SS Kim DY Enhanced performance of inverted polymer solar cells with cathode interfacial tuning via water-soluble polyfluorenes Appl Phys Lett 2010 97 223305 10.1063/1.3522893

[B35] Belghachi A Perimeter recombination in thin film solar cells J Comput Electron 2007 6 279 283 10.1007/s10825-006-0123-5

[B36] Kim HS Lee JW Yantara N Boix PP Kulkarni SA Mhaisalkar S Gräetzel M Park NG High efficiency solid-state sensitized solar cell-based on submicrometer rutile TiO_2_ nanorod and CH_3_NH_3_PbI_3_ perovskite sensitizer Nano Lett 2013 13 2412 2417 10.1021/nl400286w 23672481

[B37] Jeng JY Chiang YF Lee MH Peng SR Guo TF Chen P Wen TC CH_3_NH_3_PbI_3_ perovskite/fullerene planar-heterojunction hybrid solar cells Adv Mater 2013 25 3727 3732 10.1002/adma.201301327 23775589

